# Construction of the prognostic nomogram and treatment recommendation in patients with mixed endometrial carcinoma treated with hysterectomy

**DOI:** 10.17305/bb.2024.10754

**Published:** 2024-07-08

**Authors:** Luyao Kang, Gaili Ji, Nan Zhang, Jie Meng, Duan Liu, Hongyu Li

**Affiliations:** 1 Department of Gynecologic Oncology, The Third Affiliated Hospital of Zhengzhou University, Zhengzhou, China

**Keywords:** Mixed endometrial carcinoma (MEC), Surveillance, Epidemiology, and End Results (SEER), nomogram, prognosis, treatment

## Abstract

Mixed endometrial carcinomas (MECs) account for approximately 3%–10% of all endometrial carcinomas (ECs). These are defined as a combination of two or more distinct histologic subtypes, with at least one being a type II tumor that constitutes at least 5% of the overall tumor. However, the associated prognostic factors and treatment of MECs remain unclear. The study aimed to identify the independent prognostic factors of MEC patients treated with hysterectomy and to explore the optimal treatment modalities for overall survival (OS) and cancer-specific survival (CSS). Using the Surveillance, Epidemiology, and End Results (SEER) database, a total of 12,848 MEC patients treated with hysterectomy were screened. Independent prognostic factors were identified by Cox regression analysis and used to construct the nomogram. The concordance indices (C-indices) of OS and CSS were 0.807 and 0.834 in the training set. Validation of the nomogram revealed that the receiver operating curve (ROC) maintained good discrimination, the decision curve analysis (DCA) had a high net benefit rate, and the calibration curves showed high consistency. Patients were grouped by the nomogram formula and the number of positive regional lymph nodes (NPR-Lymph node) to evaluate the therapeutic outcomes of chemotherapy, radiotherapy, neoadjuvant treatment, and lymph node operation. Survival analysis revealed that chemotherapy could improve the prognosis for OS and CSS in the high-risk group and in the group with NPR-Lymph node counts above 1 (*P* < 0.05). Radiotherapy was associated with better OS and CSS in the intermediate-risk and high-risk groups, and in the group with NPR-Lymph node counts above 0 (*P* < 0.05). Lymphadenectomy was found to prolong OS and CSS in the high-risk group (*P* < 0.05), while neoadjuvant treatment did not prolong OS and CSS in any group. Thus, in this study, the nomogram for MEC patients treated with hysterectomy was successfully built and validated which could effectively predict the prognosis and identify at-risk population to guide clinical decision making. The NPR-Lymph node was identified as a potentially strong prognostic indicator with good clinical value.

## Introduction

Endometrial carcinoma (EC) is the second most common tumor of the female reproductive system [1]. It has traditionally been classified into two types based on clinical characteristics and pathology: types I and II [2]. Type I tumors are estrogen dependent and associated with endometrial hyperplasia, whereas type II tumors are estrogen independent and associated with endometrial atrophy [3]. Although this classification is widely used, diagnosing mixed endometrial carcinomas (MECs) still remains challenging [4, 5]. They are defined as a combination of two or more distinct histologic tumor subtypes, one of which must be a type II tumor, such as serous carcinoma or clear cell carcinoma [6]. The most common combination is an admixture of endometrioid carcinoma and serous carcinoma. According to the WHO criteria, diagnosing MECs requires that the serous or clear cell carcinoma component accounts for at least 5% of the overall tumor [7].

Recent studies have shown that even a small portion of serous or clear cell carcinoma can lead to aggressive characteristics similar to those of pure serous or clear cell carcinoma [8, 9]. While there is limited literature on the prognosis and treatment of MECs, highlighting the importance of investigating MEC patients to improve survival probability. As total hysterectomy is considered the primary treatment for EC [10, 11], this study focused on patients with MEC who underwent hysterectomy to investigate the prognostic factors and optimal treatment modalities for improving overall survival (OS) and cancer-specific survival (CSS) outcomes.

## Materials and methods

### Study population

**Figure 1.**
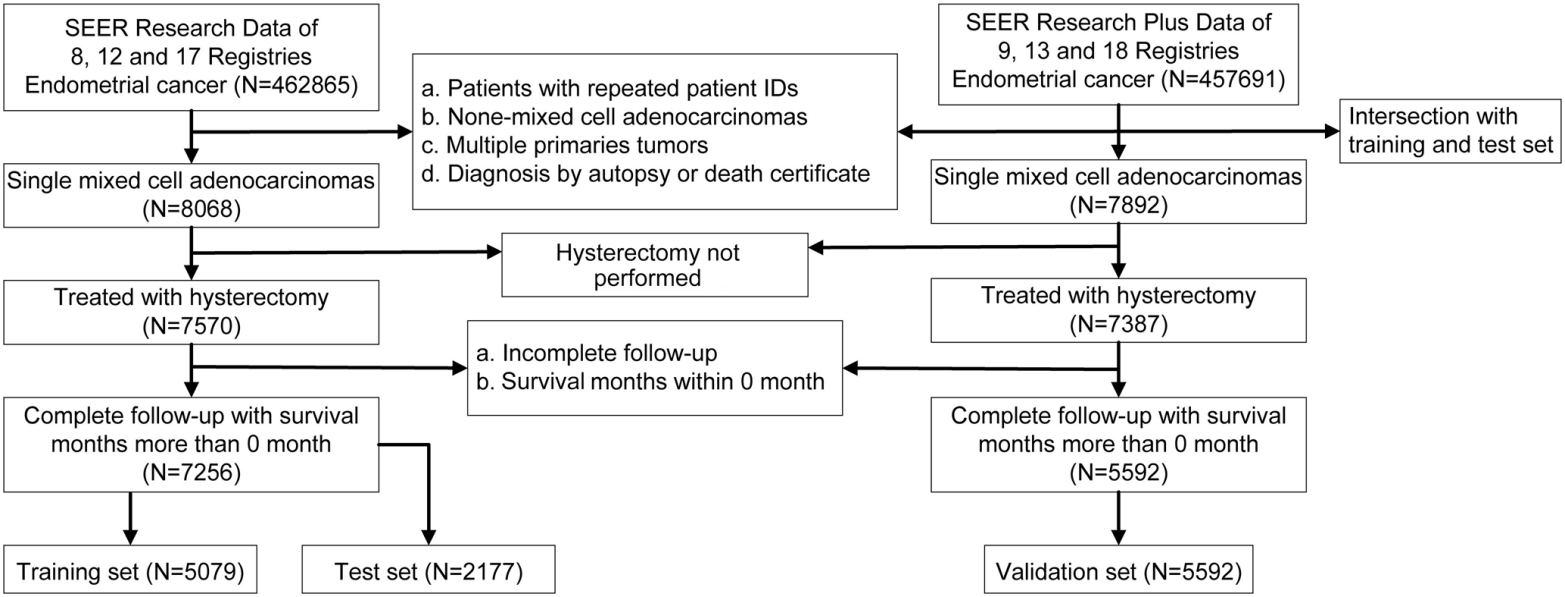
**Scheme of mixed endometrial carcinoma patients screening process.** SEER: Surveillance, Epidemiology, and End Results.

We used SEER*Stat software [version 8.4.3; Surveillance, Epidemiology, and End Results (SEER) research data (from 8, 12, and 17 registries) of the November 2022 submission and SEER research plus data (from 9, 13, and 18 registries) of the November 2020 submission] to identify patients with MEC. The tumor information in the SEER database from different registries is unified and standardized by SEER*Stat software. Women meeting the following criteria were included in the study: 1) primary site: ICD-O-3 of C54.1, endometrium; 2) unique patient ID; 3) histology of 8323/3; 4) single primary tumor; 5) diagnosis not by autopsy or death certificate; 6) hysterectomy performed; and 7) complete follow-up with survival months more than a month. As for the validation set, patients who were intersected with the training and test sets were excluded from the study. Through the inclusion and exclusion criteria (Figure 1), 12,848 eligible MEC patients were finally screened.

### Study variables

Clinical variables extracted from the SEER database include age at diagnosis (y), race, marital status, tumor size (mm), grade, SEER-Stage, AJCC-Stage, AJCC-T, NPR-Lymph node (number of positive regional lymph node), distant metastasis, peritoneal cytology, lymph node operation, radiotherapy, chemotherapy, and neoadjuvant treatment.

### Ethical statement

The clinical data in this retrospective study were collected from the publicly available SEER database, so there were no local or national ethical issues, and informed consent was not required.

### Statistical analysis

The 7256 eligible patients from SEER research data (8, 12, and 17 registries) of the November 2022 submission were divided into a training set (*N* ═ 5079) and a test set (*N* ═ 2177) at a ratio of 7:3. The validation set (*N* ═ 5592) was extracted from SEER research plus data (9, 13, and 18 registries) of the November 2020 submission. For continuous variables, including age at diagnosis (Figure S1A–S1C), tumor size (Figure S1D–S1F), and NPR-Lymph node (Figure S1G–S1I), the optimal cut-off values were calculated by X-tile software [12] based on OS using the data from the training set and test set. And the prognosis of patients was closely related to the optimal cut-off. Then, the age at diagnosis, tumor size, and NPR-Lymph node were converted to categorical variables. The chi-square test was used to compare the differences in baseline characteristics between the training and test sets. Univariate Cox regression analysis was used to analyze the correlation between variables and survival outcomes. Variables with significance (*P* < 0.05) of OS and CSS were separately included in the multivariate Cox regression analysis. The common multivariate Cox proportional hazard results were used as the basis for the construction and validation of the nomogram. The data for the validation of the nomogram included the training set, test set, and validation set. Harrell’s concordance index (C-index) [13] was calculated for the nomogram, which was used to evaluate the predictive ability of the model. The C-index ranges between 0.5 and 1.0; 0.5 indicates a completely random model with no predictive effect, and the closer the C-index is to 1, the more accurate it is. The receiver operating characteristic (ROC) curve and time-dependent area under the curve (AUC) were used to quantify the discrimination performance and diagnostic value between the nomogram model and other models of OS and CSS in one, three, five, and ten years. The clinical utility of the decision curve analysis (DCA) between the nomogram model and other models was used to observe the net benefit of the nomogram. Meanwhile, the Hosmer–Lemeshow goodness of fit test was used for drawing the calibration curve.

The survival time was defined as the duration from diagnosis to either death or the last follow-up. Patients were categorized into low-, intermediate-, and high-risk groups based on the optimal cut-off value determined by X-tile software using the risk score calculated from the nomogram formula of OS and CSS for each individual. The treatment effects of chemotherapy, radiotherapy, neoadjuvant treatment, and lymph node operation for the prognosis of the MEC patients treated with hysterectomy were analyzed by Kaplan–Meier curve and Log-Rank test. Additionally, patients were stratified based on the NPR-Lymph node using X-tile software to evaluate its influence on the treatment outcomes of chemotherapy, radiotherapy, and neoadjuvant treatment through survival analysis.

All statistical analyses were performed using the packages of R 4.3.3 (https://www.r-project.org/), containing “randomForestSRC” [14], “ezcox” [15], “survival” [16], “survminer” [17], “rms” [18], “regplot” [19], “riskRegression” [20], “cmprsk” [21], “QHScrnomo” [22], “timeROC” [23], “tidyverse” [24], “paletteer” [25], “compareGroups” [26], “ggDCA” [27], and “tidycmprsk” [28]. A bilateral *P* value < 0.05 was considered statistically significant.

## Results

### Clinical characteristics

The 7256 eligible patients who were diagnosed with MEC from SEER research data (8, 12, and 17 registries) were randomly divided into a training set (*N* ═ 5079) and a test set (*N* ═ 2177). The ideal cut-off values for the two groups of continuous variables (age at diagnosis, tumor size, and NPR-Lymph node) were determined by X-tile software (Figure S1). All baseline clinical characteristics were considered not significantly different between the training and test set (all *P* > 0.05; Table S1).

### Construction and validation of the nomogram

**Figure 2.**
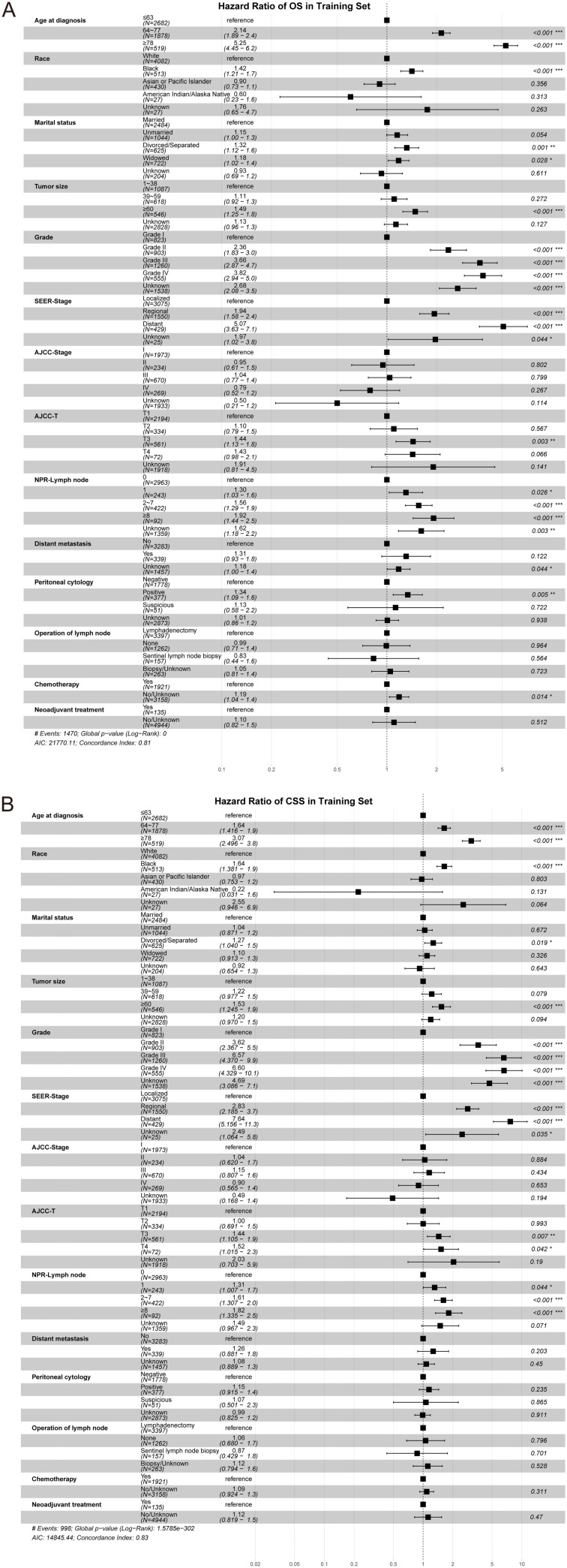
**Screening of the independent prognostic factors by multivariate Cox regression analysis in the training set.** (A) Overall survival; (B) Cancer-specific survival. SEER: Surveillance, Epidemiology, and End Results; AJCC: American Joint Committee on Cancer; NPR-Lymph node: Number of positive regional lymph node.

**Figure 3.**
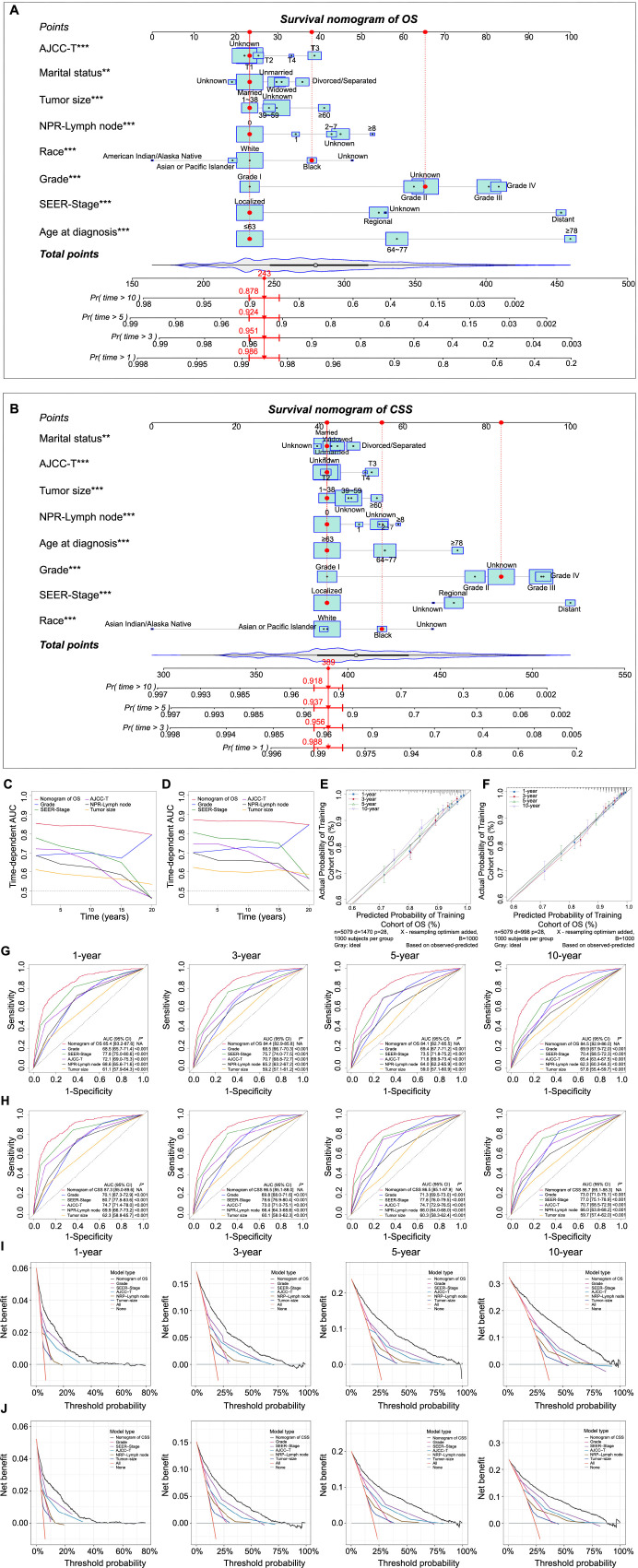
**Construction and validation of the nomogram in the training set.** The Cox regression nomogram was based on the eight independent prognostic factors, including age at diagnosis, race, marital status, grade, SEER-Stage, AJCC-T, NPR-Lymph node, and tumor size, to predict OS (A) and CSS (B) at 1-, 3-, 5-, and 10-year. The performance of the established nomogram was compared to grade, SEER-Stage, AJCC-T, NPR-Lymph node, and tumor size using the time-dependent AUC, ROC, and DCA. Time-dependent AUC for OS (C) and CSS (D). Calibration curves for the established nomogram for predicting 1-, 3-, 5-, and 10-year OS (E) and CSS (F). ROC curves for OS (G) and CSS (H) in mixed endometrial carcinoma patients at 1-, 3-, 5-, and 10-year. DCA curves for OS (I) and CSS (J). The *x*-axis shows the threshold probability, and the *y*-axis measures the net benefit. *P: Other models vs nomogram model; OS: Overall survival; CSS: Cancer-specific survival; SEER: Surveillance, Epidemiology, and End Results; AJCC: American Joint Committee on Cancer; NPR-Lymph node: Number of positive regional lymph node; AUC: Area under the curve; CI: Confidence interval; ROC: Receiver operating curve; DCA: Decision curve analysis.

Independent prognostic factors of CSS and OS were screened based on univariate (training set: Table S2; test set: Table S3) and multivariate Cox regression analyses in the training set (OS: Figure 2A; CSS: Figure 2B). The results of the multivariate Cox regression analysis in the test set are shown in Figure S2 (OS: Figure S2A; CSS: Figure S2B). The nomogram model (OS: Figure 3A; CSS: Figure 3B) was constructed using the commonly screened independent predictors of OS and CSS (age at diagnosis, race, marital status, tumor size, grade, SEER-Stage, AJCC-T, and NPR-Lymph node). Each category within these variables was given a score. By adding up the scores for each variable and placing them on the total points scale, the predicted survival probability of OS and CSS can be easily obtained for the patients.

The other 5592 eligible MEC patients were extracted from SEER research plus data (9, 13, and 18 registries) and used for external verification. Table S4 displays the clinical characteristics of the validation set. For the training set, the C-indexes of OS and CSS were 0.807 and 0.834. For the test set, the C-indexes of OS and CSS were 0.789 and 0.830. For the validation set, the C-indexes of OS and CSS were 0.810 and 0.838. The ROC curves of the nomogram, grade, SEER-Stage, AJCC-T, NPR-Lymph node, and tumor size for OS (training set: Figure 3G; test set: Figure S3A; validation set: Figure S4A) and CSS (training set: Figure 3H; test set: Figure S3B; validation set: Figure S4B) were utilized to assess the predictive ability of the nomogram. The results showed that the prognostic performance of the nomogram was significantly better than other prognostic factors, having an AUC greater than 0.8. The time-dependent AUC illustrated the stable diagnostic value of the nomogram for OS and CSS (training set: Figure 3C and 3D; test set: Figure S3E and S3F; validation set: Figure S4E and S4F). DCA curves of OS (training set: Figure 3I; test set: Figure S3C; validation set: Figure S4C) and CSS (training set: Figure 3J; test set: Figure S3D; validation set: Figure S4D) revealed a good net benefit in clinical practice. At the same time, calibration curves of the nomogram model showed excellent agreement between predictions and actual observations for OS and CSS at one, three, five, and ten years (training set: Figure 3E and 3F; test set: Figure S3G and S3H; validation set: Figure S4G and S4H).

### Subgroup Kaplan–Meier survival analysis

**Figure 4.**
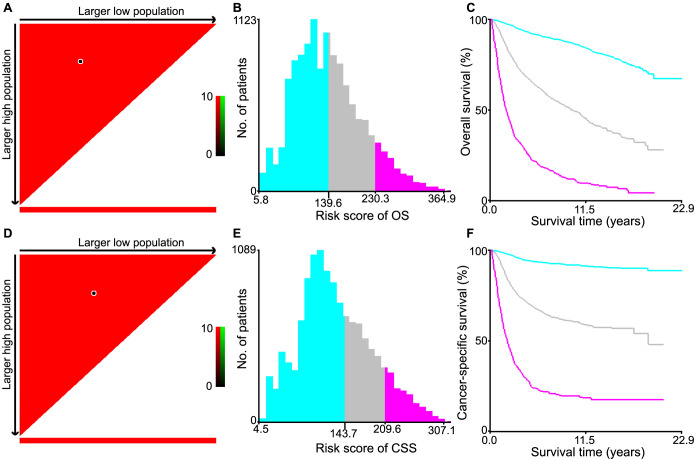
**Calculation of the optimal cutoff values of the risk score.** The optimal cutoff values of the risk score for OS (A–C) and CSS (D–F) were calculated by the X-tile software using the data of the training, test, and validation sets. The dark dots in the X-tile plots were the sites according to the highest χ2 value defined by Kaplan–Meier survival analysis and the Log-Rank test in the established nomogram based on OS (A) and CSS (D). Histograms of the risk score based on OS (B) and CSS (E) in low-, intermediate-, and high-risk groups according to the optimal cutoff values. In Kaplan–Meier survival analysis, cyan bars represent the low-risk group, gray bars represent the intermediate-risk group, and purple bars represent the high-risk group. Kaplan–Meier survival analysis of OS (C) and CSS (F) by the optimal cutoff values. OS: Overall survival; CSS: Cancer-specific survival.

**Figure 5.**
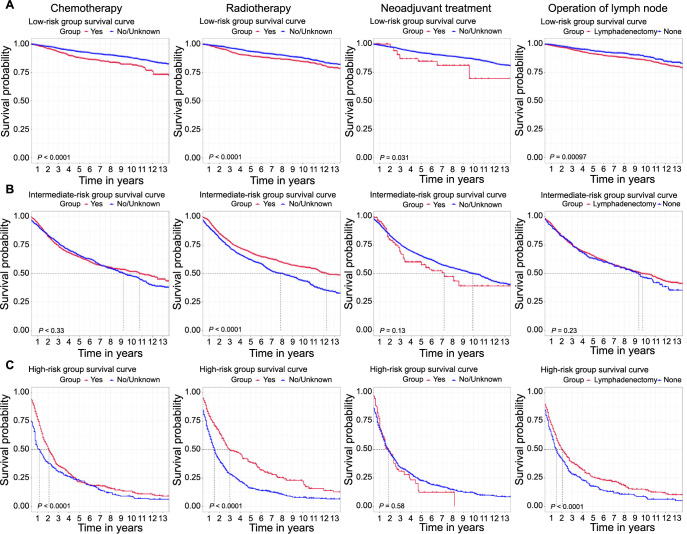
**Kaplan–Meier survival curves of MEC patients for OS by risk score (low-risk, intermediate-risk, and high-risk) with chemotherapy, radiotherapy, neoadjuvant treatment, and lymph node operation in the total patients (training, test, and validation set).** Lymph node operation with sentinel lymph node biopsy and biopsy/unknown were excluded due to small sample size. (A) Low-risk MEC patients; (B) Intermediate-risk MEC patients; (C) High-risk MEC patients. MEC: Mixed endometrial carcinoma; OS: Overall survival.

MEC patients were classified into the low-, intermediate-, and high-risk groups according to the risk score generated from the nomogram formula by X-tile software (OS: Figure 4A–4C; CSS: Figure 4D–4F). The Kaplan–Meier survival analysis based on the above classification was separately constructed for OS (Figure 5A–5C) and CSS (Figure S5A–S5C). As for the low-risk group of MEC patients for OS (Figure 5A) and CSS (Figure S5A), the absence of chemotherapy, radiotherapy, neoadjuvant treatment, and lymph node operation showed better survival outcomes. As for the intermediate-risk group (OS: Figure 5B; CSS: Figure S5B), radiotherapy was associated with better OS and CSS, while other treatments for the MEC patients had no statistical significance (*P* > 0.05). As for the high-risk group for OS (Figure 5C) and CSS (Figure S5C), chemotherapy, radiotherapy, and lymphadenectomy could improve the prognosis of MEC patients, whereas neoadjuvant treatment did not prolong OS and CSS in any group.

**Figure 6.**
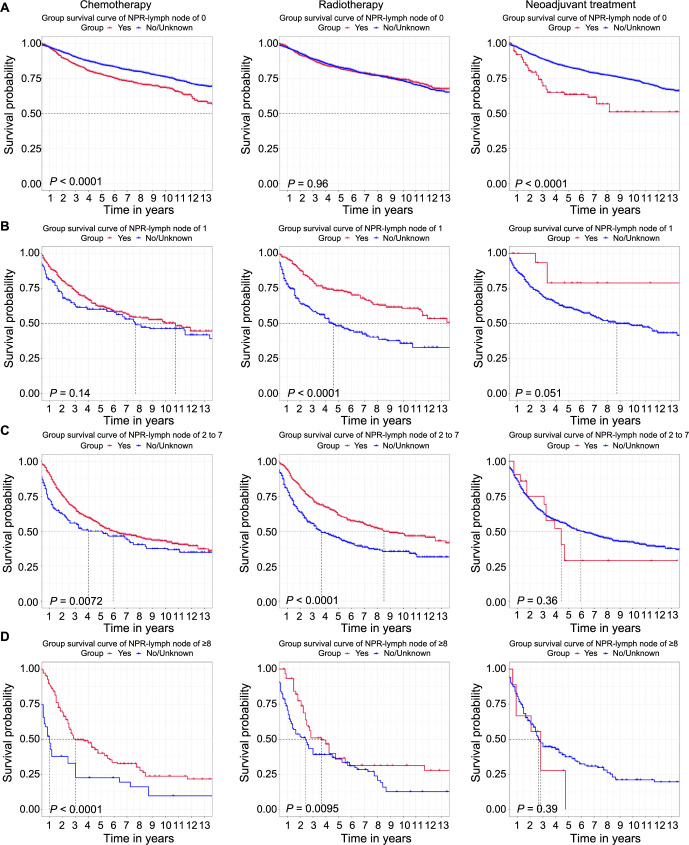
**Kaplan–Meier survival curves of MEC patients for OS by the NPR-Lymph node with chemotherapy, radiotherapy, and neoadjuvant treatment in the total patients (training, test, and validation set).** (A) MEC patients with NPR-Lymph node of 0; (B) MEC patients with NPR-Lymph node of 1; (C) MEC patients with NPR-Lymph node of 2–7; (D) MEC patients with NPR-Lymph node of ≥8. NPR-Lymph node: Number of positive regional lymph node; MEC: Mixed endometrial carcinoma; OS: Overall survival.

We also grouped the total patients based on the NPR-Lymph node by X-tile software for OS (Figure 6A–6D) and CSS (Figure S6A–S6D) to assess its influence on the treatment effect of chemotherapy, radiotherapy, and neoadjuvant treatment. As for the group of NPR-Lymph node of 0 (OS: Figure 6A; CSS: Figure S6A), the absence of chemotherapy and neoadjuvant treatment was associated with better OS and CSS. However, radiotherapy had ambiguous results, which had no statistical significance for OS, while MEC patients who had no radiotherapy had a better prognosis for CSS. As for the group of NPR-Lymph node of 1 for OS (Figure 6B) and CSS (Figure S6B), only radiotherapy could improve the prognosis. As for the group of NPR-Lymph node above 1 including the NPR-Lymph node of 2–7 group (OS: Figure 6C; CSS: Figure S6C) and the NPR-Lymph node of ≥8 group (OS: Figure 6D; CSS: Figure S6D), chemotherapy and radiotherapy were found to be associated with prolonged OS and CSS. In contrast, neoadjuvant treatment did not lead to a significant extension of OS and CSS in any of the groups.

## Discussion

MECs account for approximately 3%–10% of all ECs [29, 30]. The pathogenesis of MECs remains to be fully elucidated. Recent molecular genetic studies suggest that some may arise through completely unrelated oncogenic mechanisms (collision tumor) while others may share a common oncogenic origin, such as progression from one histologic type to another, divergence from a common progenitor into different histologic types, and a single tumor histologic type that focally displays a variant morphology that mimics a different histologic type [5, 6, 31–33]. The unclear pathogenesis reveals the possible diverse biological behavior of MECs [34–38].

With the successful proposal of TCGA EC molecular subtypes [39] and the development of Proactive Molecular Risk Classifier in Endometrial Cancer (ProMisE) [40], the integration of histopathological features and molecular information provides a more suitable way for better classification diagnosis, prognosis evaluation, and therapeutic applications of EC [41, 42]. However, applying those principles to MEC with evidence of multiple molecular classifiers is full of challenges, especially in cases where the abnormality is restricted to one component, which requires sequencing of both components separately to ensure accurate patient risk stratification and treatment [33].

Some studies have suggested that when a minor part of an EC is composed of a serous carcinoma component, the patient has the same prognosis and risk for metastases as patients with pure serous carcinoma, having the potential to adversely influence the survival of the patient [38, 43]. However, there is a scarcity of literature regarding the prognosis and treatment of MECs. In this study, through the analysis of MEC patients treated with hysterectomy, we identified possible prognostic factors and explored the therapeutic impact of chemotherapy, radiotherapy, neoadjuvant treatment, and lymph node operation in an effort to enhance patient prognosis to the greatest extent possible.

The nomogram is a multivariable prognostic model that can integrate diverse prognostic factors and is commonly used to precisely evaluate the probability of individual endpoint events in patients [44–46]. In this study, univariate and multivariate Cox regression analyses were performed to identify the independent prognostic factors of OS and CSS for the construction and validation of the nomogram. Factors that showed consistent statistical significance in both univariate and multivariate Cox regression analyses for OS and CSS were considered potential prognostic factors and were incorporated into the nomogram construction and validation process, which included age at diagnosis, race, marital status, tumor size, grade, SEER-Stage, AJCC-T, and NPR-Lymph node. Tumor size and AJCC-T stage are internationally recognized as important risk factors for EC [47, 48]. As for the SEER-Stage of MEC patients, we found the prognosis of localized patients was far better than the regional and distant patients. The prognostic value of SEER-Stage exceeded the clinical stage of AJCC-Stage. Recent articles have demonstrated that the NPR-Lymph node is potentially a stronger prognostic indicator for EC which is closely related to disease recurrence and mortality [49–51]. In this study, the data indicated that the presence of any positive regional lymph node was associated with an increased risk of mortality in patients with MEC, regardless of OS or CSS.

The ROC curve, DCA curve, and calibration curve were used to verify the clinical utility and predictive value of the nomogram model, demonstrating its superior performance compared to grade, SEER-Stage, AJCC-T stage, NPR-Lymph node, and tumor size. In practice, whether in the training set, test set, or validation set, the AUC values of the nomogram for OS and CSS consistently exceeded 80% and the time-dependent AUC provided proof for the stability of the AUC, which confirmed the good predictive ability and diagnostic value of the nomogram. The results of the DCA curves also showed the robust clinical value of the nomogram. At the same time, the calibration curve was used to assess the prediction accuracy for OS and CSS at one, three, five, and ten years, which was in good consistency with the actual observational results.

To assess the treatment outcomes for MEC patients receiving therapies other than surgery, which included chemotherapy, radiotherapy, neoadjuvant treatment, and lymph node operation in detail, we categorized the MEC patients treated with hysterectomy into low-, intermediate-, and high-risk groups by the nomogram formula. Based on the above classification, the results showed that when they were in the high-risk group, adjuvant treatment and lymphadenectomy were necessary for them. When they were in the intermediate-risk group, the adjuvant treatment should be under multifactorial evaluation, and the data revealed that radiotherapy should be implemented. Additionally, we grouped MEC patients treated with hysterectomy by the NPR-Lymph node to assess the therapeutic outcome of chemotherapy, radiotherapy, and neoadjuvant treatment. Subgroup survival analysis based on NPR-Lymph node demonstrated significant clinical utility. Once the regional lymph node was positive, the adjuvant treatment should be under consideration. When the NPR-Lymph node was 1, radiotherapy should be considered, and when the NPR-Lymph node was above 1, chemotherapy and radiotherapy should both be taken into account.

There were some limitations in this study. Firstly, the retrospective nature of the study poses significant limitations. Retrospective cohort studies are vulnerable to selection bias, recall bias, and unknown confounding variables, which can undermine the accuracy of the findings. Secondly, the SEER database did not contain detailed chemotherapy information for the use of the targeted drugs, which are of great importance in the prognosis of MECs. Thirdly, due to the population included in this study being American white people with MEC, the model constructed might not be extended to other populations. Fourthly, although the nomogram received internal and external validation, further validation studies including prospective and retrospective studies in larger and more diverse patient populations are needed to evaluate the applicability of the prognostic nomogram in different demographics and healthcare settings to provide stronger evidence for clinical application. Finally, because of a lack of information about molecular subtypes of MECs in the SEER database, more clinical practice should be carried out to explore the prognostic factors associated with different molecular subtypes of MEC in the future.

## Conclusion

The nomogram for MEC patients treated with hysterectomy was successfully built and validated. It could effectively predict the prognosis and screen the risk population to guide clinical decision making. The NPR-Lymph node was a potentially strong prognostic indicator with a good clinical value.

## Supplemental data

Supplementary data are available at the following link: https://www.bjbms.org/ojs/index.php/bjbms/article/view/10754/3361

## Data Availability

Publicly available datasets were analyzed in this study. These data can be found in https://seer.cancer.gov/data-software/.
